# Bayesian function registration with random truncation

**DOI:** 10.1371/journal.pone.0287734

**Published:** 2023-07-07

**Authors:** Yi Lu, Radu Herbei, Sebastian Kurtek

**Affiliations:** 1 Mathematics and Computer Science Department, Drew University, Madison, New Jersey, United States of America; 2 Department of Statistics, The Ohio State University, Columbus, Ohio, United States of America; Shanxi University, CHINA

## Abstract

In this work, we develop a new set of Bayesian models to perform registration of real-valued functions. A Gaussian process prior is assigned to the parameter space of time warping functions, and a Markov chain Monte Carlo (MCMC) algorithm is utilized to explore the posterior distribution. While the proposed model can be defined on the infinite-dimensional function space in theory, dimension reduction is needed in practice because one cannot store an infinite-dimensional function on the computer. Existing Bayesian models often rely on some pre-specified, fixed truncation rule to achieve dimension reduction, either by fixing the grid size or the number of basis functions used to represent a functional object. In comparison, the new models in this paper randomize the truncation rule. Benefits of the new models include the ability to make inference on the smoothness of the functional parameters, a data-informative feature of the truncation rule, and the flexibility to control the amount of shape-alteration in the registration process. For instance, using both simulated and real data, we show that when the observed functions exhibit more local features, the posterior distribution on the warping functions automatically concentrates on a larger number of basis functions. Supporting materials including code and data to perform registration and reproduce some of the results presented herein are available online.

## Introduction

Advances in data collection technology have made functional data prevalent in various applied domains including biology, biometrics, medicine, computer vision, bioinformatics, and many others. This, in turn, has prompted rapid development of functional data analysis (FDA) methods for estimation, alignment, summarization, and statistical modeling (and inference) for such data. In this work, we specifically focus on the problem of Bayesian model-based alignment of two or more functions, termed pairwise and multiple registration, respectively. In particular, we elucidate the challenges arising from nonlinearity and infinite-dimensionality of the representation spaces on which observation and prior models must be defined.

We consider the task of registration of real-valued functions defined on a subinterval of the real line. The goal of registration is to separate two sources of variability in functional data termed amplitude (*y*-axis variation) and phase (*x*-axis variation or time warping), and our aim is to temporally align (or warp) a set of functions, such that the amplitude variation in the observed data is comparable (i.e., amplitude features like local extrema occur at the same time along the *x*-axis, across all functions). The phase variation is then captured by a set of time warping functions that achieve the alignment. Formal definitions of amplitude, phase and the registration problem that are considered in this work are provided in subsequent sections.

**Fig 1:**
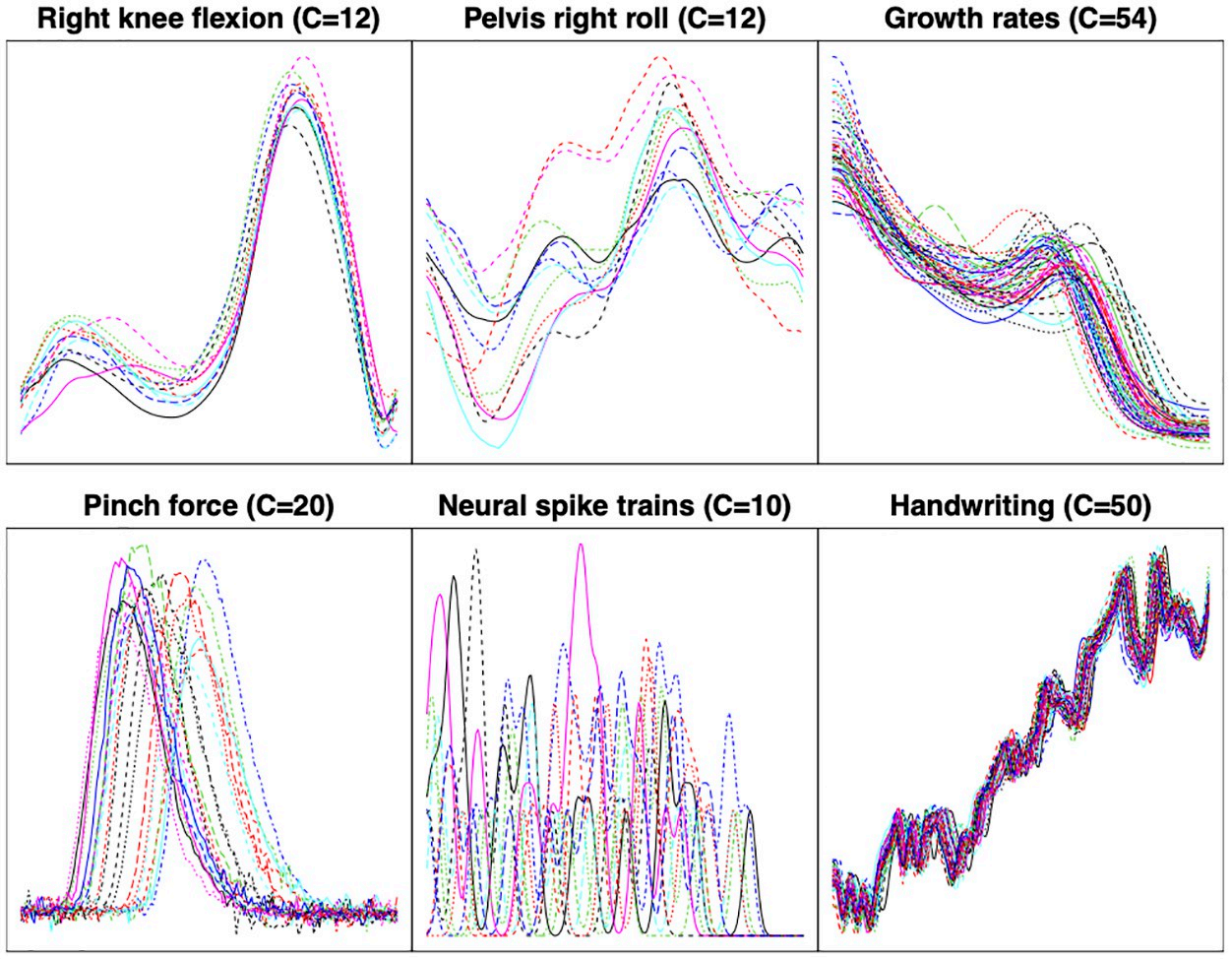
Examples of the types of functions we consider. The value *C* represents the number of functions in each dataset.

Real data examples of the types of functions we consider are plotted in Fig 1. To motivate the problem of function registration, we consider the widely-used Berkeley growth study dataset [1]. The data is comprised of height measurements for boys and girls recorded from age 1 to age 18. To discover patterns of growth such as growth spurts, it is often preferable to analyze growth rate functions, i.e., the time derivative of the height measurement functions, since periods of fast/slow growth result in local extrema. The growth rate functions for 54 girls are displayed in the third panel, top row of Fig 1. It is clear that the functions have very similar shapes and exhibit one peak near the middle of the domain, corresponding to the pubertal growth spurt. However, the pubertal growth spurt does not occur at the same time across all growth rate functions since different children go through puberty at different times. Thus, it becomes necessary to register the growth rate functions prior to statistical analysis such that the amplitude variability (magnitude of pubertal growth spurts) and phase variability (timing of pubertal growth spurts) are separated.

To enhance statistical analysis, registration of functional data has been utilized in a wide range of applications including biomechanical data [2–4], handwriting samples [5, 6], gene expression and proteomics data [7, 8], neural spike trains [9], and gait data [10, 11]. Traditionally, function registration is formulated as an optimization problem under a specific optimality criterion, preferably a metric. We refer readers to standard FDA textbooks (e.g., [12, 13]) for an overview of the many approaches that have been proposed. Recently, model-based or probabilistic frameworks have become popular in formulating the registration problem. Specifically, the Bayesian modeling paradigm provides considerable flexibility as it allows the user to specify a prior distribution over the phase parameter space. Additionally, it yields a principled approach for a more comprehensive exploration of the phase parameter space and provides quantified uncertainty measures via the posterior distribution.

The literature includes multiple different Bayesian formulations of function registration. Telesca and Inoue [7] use the B-spline basis to model functional parameters; Claeskens et al. [14] decompose time warping functions into so-called warping component functions or *warplets*. Cheng et al. [15], Bharath and Kurtek [16], and Matuk et al. [17] use Dirichlet priors for the time (increments of) a warping function. Finally, Earls and Hooker [18], Kurtek [19], and Lu et al. [11] use Gaussian process priors on a transformed warping parameter space. We refer the reader to Matuk et al. [20] for a general overview of Bayesian registration methods. As evident, the main differences among the aforementioned methods are in the specification of the observation model, the prior model on phase, and the algorithms used for parameter space exploration; we discuss benefits and drawbacks of the different choices later as we introduce the proposed framework. Some of the methods (e.g., [16]) are additionally able to incorporate information on landmarks (predetermined, user-defined points of interest) into the registration problem.

This work extends and improves the Bayesian models proposed in [11] to enable full exploration of the functional parameter space. In [11], the authors specify a Gaussian process prior over the infinite-dimensional phase parameter space and represent time warping functions using a sequence of basis functions. However, at the implementation stage, the dimension of the parameter space is reduced by choosing a *fixed* number of basis functions. Thus, the resulting model is not truly infinite-dimensional and is able to explore only a small subset of the underlying parameter space. In practice, the main disadvantage of such a formulation is that the dimension reduction is performed a priori and is thus not informed by the data. Furthermore, it is generally not obvious how many basis functions are needed to achieve satisfactory registration results.

To remedy these issues, instead of using a fixed truncation, we allow the truncation to be *random*. This is done by randomizing either (i) the *number* of basis functions or (ii) *which* basis functions are used. We incorporate this random truncation as a separate parameter, leading to nonparametric, infinite-dimensional models in the sense that the prior distributions are assigned on the entire functional phase parameter space. The proposed models with random truncation have three advantages. First, the level of smoothness of the functional phase parameter is informed by the data, thus avoiding potential mis-specifications of the number of basis functions. As we will show, under-specification of the number of basis functions can lead to poor registration results. Second, one can flexibly incorporate prior beliefs or desired constraints on the shape of the functional phase parameter. For instance, how much shape-alteration occurs in the observed functions can be controlled by the prior on the number of basis functions. Third, our model allows one to make inference on the random truncation parameter, which can provide additional information about the shapes of the functions in the data. For instance, the posterior tends to keep a larger number of basis functions for the phase parameter when the observed functions exhibit a lot of local features that must be registered. Following ideas from [11, 21, 22], we develop algorithms that allow efficient sampling from the posterior distribution of both the functional parameter and the random truncation parameter. A key designing principle for our model is to treat the phase parameter space as infinite-dimensional and to allow the data to dictate the amount of dimension reduction that is needed.

The rest of the paper is organized as follows. We first introduce the statistical problem of function registration, focusing on relevant function spaces, in Section Problem Formulation and Function Spaces of Interest. The proposed Bayesian registration models are formally specified in Sections Pairwise Bayesian Registration Model with Random Basis Truncation and Multiple Function Bayesian Registration Model with Random Basis Truncation. In Sections Simulation Study and Applications, we demonstrate the proposed method on a pairwise simulation study and several real datasets.

## Problem formulation and function spaces of interest

We formulate the task of function registration as a statistical problem by defining (i) the data and the observation space, and (ii) the parameter(s) and their corresponding representation spaces. As will be seen, it is essential to transform both the observation space and the parameter space. The transformations we adopt here are developed in [23, 24] in the context of function registration and have been utilized for registration models in a host of recent manuscripts, including [11, 17–19, 25, 26]. A comprehensive discussion of these transformations can be found in [13]. Here, for brevity, we will only introduce the relevant notation and briefly state the transformations. We refer the reader to the aforementioned references for more details.

We start with the simpler case of *pairwise* registration, where two real valued functions, *f*_1_ and *f*_2_, are observed. Without loss of generality, we assume that the domain on which the functions are observed is [0, 1]. In this scenario, these two functions are regarded as *data* with the corresponding *observation space* F={f:[0,1]↦R|fisabsolutelycontinuous}. Suppose our goal is to register *f*_2_ to *f*_1_. This is achieved by finding a *warping function* *γ* such that *f*_2_ ∘ *γ* and *f*_1_ are aligned. The role of *γ* is to warp the domain of *f*_2_ so that the amplitude variation of *f*_2_ is retained, but its phase variation is altered (ideally to match that of *f*_1_). The amount of alteration, which quantifies the difference in phase variation between *f*_1_ and *f*_2_, is captured by *γ*. The warping function *γ* is regarded as the *parameter* with the corresponding *parameter space* Γ = {*γ* : [0, 1] ↦ [0, 1] | *γ*(0) = 0, *γ*(1) = 1, 0 < *γ*′ < ∞}. In the next two paragraphs, we separately describe the transformations carried out on the (1) observation (data) space, and (2) parameter space. More details on these transformations can be found in the S1 File.

**Observation (data) space**. For f∈F, we use the *square-root velocity* transformation

$$\begin{array}{c} Q\left(f\right)\left(t\right)=\text{sign}\left(f^{\prime }\left(t\right)\right)\sqrt{|f^{\prime }\left(t\right)|}\;\equiv q\left(t\right), \end{array}$$ (1)

where *f*′ is the derivative of *f*. The resulting function, denoted by *q* for simplicity and referred to as the square-root velocity function (SRVF), is an element of the transformed observation space Q, which is a subset of $L^{2}\left(\left[0,1\right]\right)$ [23]. The mapping *Q* is bijective up to a translation and f∈F can be recovered from q∈Q using $Q^{-1}\left(q\right)\left(t\right)=f\left(0\right)+\int_{0}^{t}q\left(s\right)\left|q\left(s\right)\right|\;ds$. The SRVF of a time warped function, f∘γ∈F, is given by

$$\begin{array}{c} Q\left(f\circ \gamma \right)\left(t\right)=Q\left(f\right)(\gamma (t))\sqrt{\gamma^{\prime }\left(t\right)}\equiv \left(q,\gamma \right)\left(t\right). \end{array}$$ (2)

Note that this is not the same as function composition *q* ∘ *γ*, because of the additional term $\sqrt{\gamma^{\prime }}$; for brevity, we denote this quantity by (*q*, *γ*).

**Parameter space**. For *γ* ∈ Γ, we apply two transformations:

$$\begin{array}{c} Q\left(\gamma \right)\left(t\right)=\text{sign}\left(\gamma^{\prime }\left(t\right)\right)\sqrt{|\gamma^{\prime }\left(t\right)|}=\sqrt{\gamma^{\prime }\left(t\right)}\equiv \psi \left(t\right); \end{array}$$ (3)

$$\begin{array}{c} {\text{exp}}_{1}^{-1}\left(\psi \right)=\frac{\theta }{\text{sin}(\theta )}(\psi -\;\text{cos}(\theta ))\equiv g\text{,}\;\text{where}\;\theta =\;{\text{cos}}^{-1}(\int_{0}^{1}\psi \left(t\right)dt). \end{array}$$ (4)

The first transformation is the square-root velocity transformation in Eq (1) (note that *γ*′(*t*) > 0 ∀ *t*). The second transformation is the inverse exponential map for a unit sphere that allows us to linearize the space Ψ, which is a transformed representation space of the warping functions. The resulting function, denoted by *g*, belongs to a subset of a *linear space*, defined as *A* ≡ {*g* ∈ *T*_1_(Ψ)|exp_1_(*g*) > 0} (the notation *T*_1_(Ψ) refers to the tangent space of Ψ at the function 1; see S1 File for details). We can transform *g* back to *γ* via

$$\begin{array}{c} \gamma \left(t\right)=Q^{-1}\left(\psi \right)\left(t\right)=\int_{0}^{t}\psi^{2}\left(s\right)ds=\int_{0}^{t}\;{\text{exp}}_{1}^{2}\left(g\right)\left(s\right)ds, \end{array}$$

where ${\text{exp}}_{1}\left(g\right)=\;\text{cos}\left(\|g\|\right)+\frac{\;\text{sin}(\|g\|)}{\left\|g\right\|}g$ (‖⋅‖ is the $L^{2}$ norm). The warping of an SRVF, (*q*, *γ*), can now be written in terms of the function *g*, which lies in a linear space, via

$$\begin{array}{c} \left(q,\gamma \right)\left(t\right)=q\left(\gamma \left(t\right)\right)\sqrt{\gamma^{\prime }\left(t\right)}=q(\int_{0}^{t}\;{\text{exp}}_{1}^{2}\left(g\right)\left(s\right)ds)\;{\text{exp}}_{1}\left(g\right)\left(t\right). \end{array}$$ (5)

In summary, our approach is to perform statistical inference on the parameter *g* ∈ *A*, using the (SRVFs of) the observed data $q_{1},q_{2}\in Q$. Generalization to multiple function registration is straightforward. Suppose we observe *C* > 2 functions, denoted by *f*_1_, …, *f*_*C*_. The goal is to register them simultaneously to a *template* function, f*. In some applications, we can pre-specify a *known* function as the template (common choices include one of the observed functions or their point-wise mean). Alternatively, we can treat f* as another *unknown* parameter to be estimated. Registration is achieved via estimation of the warping functions, *γ*_*i*_, *i* = 1, …, *C*, corresponding to each observed function. After applying the same transformations, we treat $q_{1},\ldots ,q_{C}\in Q$ as data and *g*_1_, …, *g*_*C*_ ∈ *A* (the warping functions) and $Q\left(f^{*}\right)=q^{*}\in Q$ (the template function) as parameters.

## Pairwise Bayesian registration model with random basis truncation

In the case of pairwise registration, the data consists of two functions *f*_1_ and *f*_2_, represented via their SRVFs *q*_1_ and *q*_2_, observed on a finite grid of size *N* denoted by [*t*] = {*t*_1_, …, *t*_*N*_}. We use the notation [*t*] to denote discretization of the domain [0, 1] throughout the rest of the paper. Thus, *f*([*t*]) denotes evaluations of the function *f* at the domain points [*t*]; similarly, $\int_{0}^{\left[t\right]}h\left(s\right)ds$ denotes the *N*-dimensional vector $\left(\int_{0}^{t_{1}}h\left(s\right)ds,\ldots ,\int_{0}^{t_{N}}h\left(s\right)ds\right)$. We model the difference between *q*_1_([*t*]) and (*q*_2_, *γ*)([*t*]) by a zero-mean *N*-dimensional Gaussian distribution. The main parameter of interest is the warping function *γ* ∈ Γ represented via *g* ∈ *A*. At the implementation stage, dimension reduction is necessary, and this is achieved by an auxiliary variable *T*. Specifically, we first represent *g* by an infinite sum using basis functions. Then, we use the random variable *T* to truncate the infinite sum to a finite sum, which can be evaluated on a computer. The *truncated* version of *g* will be henceforth denoted by $\tilde{g}$.

The pair (*g*, *T*) fully specifies $\tilde{g}$ and we specify a prior distribution for $\tilde{g}$ by assigning a joint prior probability model for the pair (*g*, *T*). To that end, we use a Gaussian process to model *g* and a general distribution *τ*_*T*_ that does not depend on *g* to model *T*. We further ensure that the pair (*g*, *T*) results in a valid warping function by restricting the joint prior to the domain $B\equiv \{\left(g,T\right):\;{\text{exp}}_{1}\left(\tilde{g}\right)>0\}$. The full model is given below.

**Model 1**.

$$q_{1}\left(\left[t\right]\right)-q_{2}(\int_{0}^{\left[t\right]}\;{\text{exp}}_{1}^{2}\left(\tilde{g}\right)\left(s\right)\;ds)\cdot \;{\text{exp}}_{1}\left(\tilde{g}\right)\left(\left[t\right]\right)\;|\;g,T,\sigma_{1}^{2}∼N\left(0_{N},\sigma_{1}^{2}I_{N}\right),$$

$$g,T∼{\left\{Gaussian\left(0,C_{g}\right)\cdot \tau_{T}\right\}}_{B},$$

$$\sigma_{1}^{2}∼IG\left(shape=a,scale=b\right),$$

*where* $\tilde{g}$ *is a function of g and T*, $C_{g}$ *is a covariance operator for the Gaussian process prior*, *τ*_*T*_ *is a prior distribution for* *T*, {⋅}_*B*_ *denotes the truncation of the joint prior distribution to the set B*, *IG*(⋅, ⋅) *is the inverse-gamma distribution, and a and b are fixed constants*.

The likelihood function is then given by

$$\begin{array}{c} L\left(g,T,\sigma_{1}^{2}\;|\;q_{1},q_{2}\right)={\left(\frac{1}{\sqrt{2\pi }}\right)}^{N}{\left(\frac{1}{\sigma_{1}^{2}}\right)}^{N/2}\;\text{exp}\{-\frac{1}{2\sigma_{1}^{2}}\cdot \text{SSE}\left(\tilde{g}\right)\}, \end{array}$$ (6)

where

$$\begin{array}{c} \text{SSE}\left(\tilde{g}\right)=\sum_{i=1}^{N}{\left(q_{1}\left(t_{i}\right)-q_{2}(\int_{0}^{t_{i}}\;{\text{exp}}_{1}^{2}\left(\tilde{g}\right)\left(s\right)ds)\;{\text{exp}}_{1}\left(\tilde{g}\right)\left(t_{i}\right)\right)}^{2}. \end{array}$$ (7)

This likelihood is identical to that of [11, 19], except that the truncated parameter $\tilde{g}$ replaces the parameter *g*.

### Random truncation mechanisms

We now discuss the following two mechanisms for the prior distribution of the random truncation *T*, which is key to the proposed approach.

**(1) Random Number of Basis Functions**. In the first scenario, consider *T* ≡ *M*, where *M* is the *number* of basis functions used to represent the parameter *g*. In this case, *π*_*T*_ ≡ *π*_*M*_ is a prior distribution on the set of positive integers and $\tilde{g}\left(t\right)=\sum_{i=1}^{M}\xi_{i}b_{i}\left(t\right)$, where {*b*_*i*_}_*i* ≥ 1_ forms an orthonormal basis for *T*_1_(Ψ).

**(2) Random Indicators**. In the second scenario, we can randomly switch a basis function on and off (called a sieve prior in [22]). This is done via the random sequence {*χ*_*i*_}_*i* = 1, …, ∞_, *χ*_*i*_ ∈ {0, 1}; we refer to this sequence simply as *χ*. This sequence controls *which* basis functions are kept in the basis expansion of *g*. In other words, *T* ≡ *χ* and $\tilde{g}$ is calculated as $\tilde{g}\left(t\right)=\sum_{i=1}^{\infty }\chi_{i}\cdot \xi_{i}b_{i}\left(t\right)$. Let *M*_*max*_ be the maximum number of basis functions stored at the implementation stage, and let *M*_*on*_ be the number of active basis functions (i.e., $M_{on}=\sum_{i=1}^{M_{max}}\chi_{i}$). The domain of *χ*, denoted X, is the collection of vectors of the form $\left(\chi_{1}\in \left\{0,1\right\},\ldots ,\chi_{M_{max}}\in \left\{0,1\right\}\right)$, and *π*_*T*_ ≡ *π*_*χ*_ is a prior on X.

### Posterior distribution and sampling via Markov chain Monte Carlo

The posterior distribution is a probability measure *μ* on the product space $T_{1}\left(\Psi \right)\times \text{(domain}\;\text{of}\;T\text{)}\times R^{+}$ and is dominated by the prior measure *μ*_0_. The Radon-Nikodym derivative is given by Bayes’ formula: $\frac{d\mu }{d\mu_{0}}\left(g,T,\sigma_{1}^{2}\right)\propto L\left(g,T,\sigma_{1}^{2}|q_{1},q_{2}\right)$. We use a Metropolis-within-Gibbs algorithm to sample from the posterior distribution of $\left(g,T,\sigma_{1}^{2}\right)$. To update the posterior at each step, the algorithm iteratively draws from (i) the full conditional distribution of (*g*, *T*), and (ii) the full conditional distribution of $\sigma_{1}^{2}$. Details are given as follows.

**Sampling of (g,T)**. We first update *g*. For this purpose, we use a *Z*-mixture *pCN* proposal (see [11, 22] for details) by setting $g^{\prime }=g\sqrt{\left(1-\beta_{z}^{2}\right)}+\beta_{z}\xi$, where *g* is the current value, *ξ* is a draw from the Gaussian process prior, and *β*_*z*_ ∈ (0, 1) is a tuning parameter drawn with probability *p*_*z*_ satisfying $\sum_{z=1}^{Z}p_{z}=1$. As an example, in the case of a 2-mixture *pCN* proposal, we can draw *β*_1_ = 0.5 with probability 0.8 and *β*_2_ = 0.001 with probability 0.2, resulting in “big jump proposals” approximately 80% of the time and “very small jump proposals” approximately 20% of the time. Sampling a new function *ξ* from $Gaussian(0,C_{g})$ is done via the Karhunen-Loève expansion. We specify the covariance operator $C_{g}$ by its eigenpairs ${\left\{(b_{i},\lambda_{i}^{2})\right\}}_{i\geq 1}$, where {*b*_*i*_(⋅)} forms an orthonormal basis for *T*_1_(Ψ) and the sequence of coefficients satisfy $\sum \lambda_{i}^{2}<\infty$. While theoretically *i* = 1, …, ∞, at the implementation stage, we store a large number *M*_*max*_ of basis functions. We thus sample independent random variables $\xi_{i}∼N\left(0,\lambda_{i}^{2}\right),\;i=1,\ldots ,M_{max}$. Then, $\xi \left(t\right)=\sum_{i=1}^{M}\xi_{i}b_{i}\left(t\right)$ if truncation mechanism (1) is used, or $\xi \left(t\right)=\sum_{i=1}^{M_{max}}\chi_{i}\cdot \xi_{i}b_{i}\left(t\right)$ if truncation mechanism (2) is used.

We then independently generate a proposal, *T*′, according to a density $Q_{T}\left(\cdot |\cdot \right)$, which depends on the truncation mechanism. The pair (*g*′, *T*′) is accepted with probability 1 ∧ *ρ*, where

$$\begin{array}{c} \rho =\frac{L\left(g^{\prime },T^{\prime },\sigma_{1}^{2}\right)\cdot \pi_{T}\left(T^{\prime }\right)\cdot Q_{T}\left(T|T^{\prime }\right)}{L\left(g,T,\sigma_{1}^{2}\right)\cdot \pi_{T}\left(T\right)\cdot Q_{T}\left(T^{\prime }|T\right)}\cdot I\left\{\left(g^{\prime },T^{\prime }\right)\in B\right\}, \end{array}$$ (8)

and *π*_*T*_ is the density function (with respect to the Lebesgue measure) of *τ*_*T*_. This acceptance ratio is intuitive if distributions of *g* have densities with respect to the Lebesgue measure. In that case, the form of *ρ* follows directly from the fact that $Gaussian\left(g^{\prime };0,C_{g}\right)\cdot Q_{pCN}\left(g|g^{\prime }\right)$ is symmetric in *g* and *g*′ ($Q_{pCN}$ is the *pCN* proposal described earlier). Since a dominating Lebesgue measure does not exist on *T*_1_(Ψ), we can derive the acceptance ratio formally using the dominating prior Gaussian measure (given in the S1 File).

If truncation mechanism (1) is used, $Q_{T}\left(T|T^{\prime }\right)=Q_{M}\left(M|M^{\prime }\right)$, and we can use a *K*-step random walk proposal of the form

$$M^{\prime }=\{\begin{array}{cc} M & \;\text{w.p.}\;p_{0}\\ M\pm 1 & \;\text{w.p.}\;p_{1}\\ M\pm 2 & \;\text{w.p.}\;p_{2}\\ \vdots & \\ M\pm K & \;\text{w.p.}\;p_{K} \end{array}$$

where $p_{0}+\sum_{k=1}^{K}2p_{k}=1$. In other words, *M*′ can either stay at the current value, with probability *p*_0_, or move forward or backward up to *K* steps. This is a symmetric proposal and the Metropolis Hastings (MH) acceptance ratio in Eq (8) simplifies to

$$\begin{array}{c} \rho =\frac{L\left(g^{\prime },M^{\prime },\sigma_{1}^{2}\right)\cdot \pi_{M}\left(M^{\prime }\right)}{L\left(g,M,\sigma_{1}^{2}\right)\cdot \pi_{M}\left(M\right)}\cdot I\left\{\left(g^{\prime },M^{\prime }\right)\in B\right\}. \end{array}$$ (9)

If the truncation mechanism (2) is used, then $Q_{T}\left(T|T^{\prime }\right)=Q_{\chi }\left(\chi |\chi^{\prime }\right)$, and we can use an *on-or-off* proposal, where *χ*′ is proposed by either switching on a nonactive basis function, with probability 0.5, or switching off an active basis function, again with probability 0.5. If all of the basis functions are currently on, i.e., *M*_*on*_ = *M*_*max*_, we switch one of them off with probability 1. On the other hand, if only one basis function is on, i.e., *M*_*on*_ = 1, we switch on another basis function with probability 1. This is the form of proposal suggested in [22]. It is not a symmetric proposal and the MH acceptance ratio in Eq (8) can be written as

$$\begin{array}{c} \rho =\frac{L\left(g^{\prime },\chi^{\prime },\sigma_{1}^{2}\right)\cdot \pi_{\chi }\left(\chi^{\prime }\right)}{L\left(g,\chi ,\sigma_{1}^{2}\right)\cdot \pi_{\chi }\left(\chi \right)}\cdot a_{\chi ,\chi^{\prime }}\cdot I\left\{\left(g^{\prime },\chi^{\prime }\right)\in B\right\}, \end{array}$$ (10)

where

$$a_{\chi ,\chi^{\prime }}=\{\begin{array}{cc} \left(M_{max}-M_{on}\right)/\left(M_{on}+1\right) & \;\text{if}\;M_{on}\neq 1,M_{on}\neq M_{max},M_{on}^{\prime }=M_{on}+1\\ M_{on}/\left(M_{max}-M_{on}+1\right) & \;\text{if}\;M_{on}\neq 1,M_{on}\neq M_{max},M_{on}^{\prime }=M_{on}-1\\ (M_{max}-1)/4 & \;\text{if}\;M_{on}=1\\ M_{max}/2 & \;\text{if}\;M_{on}=M_{max}. \end{array}$$

The derivation of *a*_*χ*,*χ*′_ is given in the S1 File.

Alternatively, one can consider a symmetric, *choose-k* proposal, where *χ*′ is proposed via the following steps: (i) sample *k* ∈ {1, 2, …, *K* ≤ *M*_*max*_} (for simplicity, we set the probability for each value of *k* to be the same, but this is not required by the algorithm), (ii) randomly select *k* entries from the vector $\chi =(\chi_{1}\in \left\{0,1\right\},\ldots ,\chi_{M_{max}}\in \left\{0,1\right\})$, and (iii) propose *χ*′ by switching (on to off, off to on) all of the selected entries. This proposal is symmetric. For example, if *χ* = (0, **0**, **1**, 1) and *χ*′ = (0, **1**, **0**, 1), then $Q_{\chi }\left(\chi^{\prime }|\chi \right)=Q_{\chi }\left(\chi |\chi^{\prime }\right)=P\left(select\;2nd\;and\;3rd\;entries|k=2\right)P\left(k=2\right)$. As a result, the MH acceptance ratio for this proposal is the same as the one given in Eq (10) with *a*_*χ*,*χ*′_ = 1.

**Sampling of** $\sigma_{1}^{2}$ . To update $\sigma_{1}^{2}$, we draw directly from the conjugate inverse-gamma distribution with shape parameter $\frac{N}{2}+a$ and scale parameter $\frac{1}{2}\text{SSE}\left(\tilde{g}\right)+b$.

## Multiple function Bayesian registration model with random basis truncation

The model for multiple function registration is a direct extension of Model (1). Based on the observed functions *f*_1_, …, *f*_*C*_, represented via the SRVFs *q*_1_, …, *q*_*C*_, we aim to make inference on ${\tilde{g}}_{1},\ldots ,{\tilde{g}}_{C}$, which are determined by the pairs (*g*_1_, *T*_1_)…, (*g*_*C*_, *T*_*C*_). In addition, we treat the template function q* as a parameter and consider the same truncation mechanisms as described in the pairwise case. The truncated template function is denoted by ${\tilde{q}}^{*}$ and is determined by the pair (q*, *T*_*q*_). We assign a Gaussian process prior to q* and a prior $\tau_{T}^{*}$ to the random truncation *T*_*q*_.

**Model 2**.

$$\begin{array}{c} q_{1}(\int_{0}^{\left[t\right]}\;{\text{exp}}_{1}^{2}\left({\tilde{g}}_{1}\right)\left(s\right)ds)\;{\text{exp}}_{1}\left({\tilde{g}}_{1}\right)\left(\left[t\right]\right)-{\tilde{q}}^{*}\left(\left[t\right]\right)\;|\;q^{*},T_{q},g_{1},T_{1},\sigma_{1}^{2}∼N\left(0_{N},\sigma_{1}^{2}I_{N}\right),\\ \vdots \\ q_{C}(\int_{0}^{\left[t\right]}\;{\text{exp}}_{1}^{2}\left({\tilde{g}}_{C}\right)\left(s\right)ds)\;{\text{exp}}_{1}\left({\tilde{g}}_{C}\right)\left(\left[t\right]\right)-{\tilde{q}}^{*}\left(\left[t\right]\right)\;|\;q^{*},T_{q},g_{C},T_{C},\sigma_{1}^{2}∼N\left(0_{N},\sigma_{1}^{2}I_{N}\right),\\ \left(g_{1},T_{1}\right)∼{\left\{Gaussian\left(0,C_{g}\right)\cdot \tau_{T}\right\}}_{B},\\ \vdots \\ \left(g_{C},T_{C}\right)∼{\left\{Gaussian\left(0,C_{g}\right)\cdot \tau_{T}\right\}}_{B},\\ \left(q^{*},T_{q}\right)∼Gaussian\left(0,C_{q}\right)\cdot \tau_{T}^{*},\\ \sigma_{1}^{2}∼IG\left(shape=a,scale=b\right). \end{array}$$

The likelihood function is then given by

$$\begin{array}{c} L\left(g_{1},T_{1},\ldots ,g_{C},T_{C},q^{*},T_{q},\sigma_{1}^{2}\right)=\prod_{i=1}^{C}\{{\left(\frac{1}{\sqrt{2\pi }}\right)}^{N}{\left(\frac{1}{\sigma_{1}^{2}}\right)}^{N/2}\;\text{exp}(-\frac{1}{2\sigma_{1}^{2}}\text{SSE}\left({\tilde{g}}_{i},{\tilde{q}}^{*}\right))\}, \end{array}$$ (11)

where

$$\begin{array}{c} \text{SSE}\left({\tilde{g}}_{i},{\tilde{q}}^{*}\right)=\sum_{j=1}^{N}{\left({\tilde{q}}^{*}\left(t_{j}\right)-q_{i}(\int_{0}^{t_{j}}\;{\text{exp}}_{1}^{2}\left({\tilde{g}}_{i}\right)\left(s\right)ds)\;{\text{exp}}_{1}\left({\tilde{g}}_{i}\right)\left(t_{j}\right)\right)}^{2}. \end{array}$$ (12)

Sampling from the posterior distribution is performed in the same fashion as in the pairwise case using a Metropolis-within-Gibbs algorithm. At each step, the algorithm first updates each of the truncated warping parameters (*g*_*i*_, *T*_*i*_), *i* = 1, …, *C* sequentially via a Metropolis step. The acceptance ratio for updating any of the pairs (*g*_*i*_, *T*_*i*_) takes the same form and, for (*g*_1_, *T*_1_), is given by

$$\begin{array}{c} \;1\wedge \frac{L\left(g_{1}^{\prime },T_{1}^{\prime },\ldots ,g_{C},T_{C},q^{*},T_{q},\sigma_{1}^{2}\right)\cdot \pi_{T}\left(T_{1}^{\prime }\right)\cdot Q_{T}\left(T_{1}|T_{1}^{\prime }\right)}{L\left(g_{1},T_{1},\ldots ,g_{C},T_{C},q^{*},T_{q},\sigma_{1}^{2}\right)\cdot \pi_{T}\left(T_{1}\right)\cdot Q_{T}\left(T_{1}^{\prime }|T_{1}\right)}\cdot I\left\{\left(g_{1}^{\prime },T_{1}^{\prime }\right)\in B\right\}\\ =1\wedge \frac{\text{exp}\left\{-\frac{1}{2\sigma_{1}^{2}}\text{SSE}\left({\tilde{g}}_{1}^{\prime },{\tilde{q}}^{*}\right)\right\}\cdot \pi_{T}\left(T_{1}^{\prime }\right)\cdot Q_{T}\left(T_{1}|T_{1}^{\prime }\right)}{\text{exp}\left\{-\frac{1}{2\sigma_{1}^{2}}\text{SSE}\left({\tilde{g}}_{1},{\tilde{q}}^{*}\right)\right\}\cdot \pi_{T}\left(T_{1}\right)\cdot Q_{T}\left(T_{1}^{\prime }|T_{1}\right)}\cdot I\left\{\left(g_{1}^{\prime },T_{1}^{\prime }\right)\in B\right\}. \end{array}$$

The algorithm then updates the template (q*, *T*_*q*_) with the acceptance ratio

$$\begin{array}{c} \;1\wedge \frac{L\left(g_{1},T_{1},\ldots ,g_{C},T_{C},q^{{*}^{\prime }},T_{q}^{\prime },\sigma_{1}^{2}\right)\cdot \pi_{T}\left(T_{q}^{\prime }\right)\cdot Q_{T}\left(T_{q}|T_{q}^{\prime }\right)}{L\left(g_{1},T_{1},\ldots ,g_{C},T_{C},q^{*},T_{q},\sigma_{1}^{2}\right)\cdot \pi_{T}\left(T_{q}\right)\cdot Q_{T}\left(T_{q}^{\prime }|T_{q}\right)}\\ =1\wedge \frac{\text{exp}\{-\frac{1}{2\sigma_{1}^{2}}\sum_{i}\text{SSE}\left({\tilde{g}}_{i},{\tilde{q}}^{{*}^{\prime }}\right)\}\cdot \pi_{T}\left(T_{q}^{\prime }\right)\cdot Q_{T}\left(T_{q}|T_{q}^{\prime }\right)}{\text{exp}\{-\frac{1}{2\sigma_{1}^{2}}\sum_{i}\text{SSE}\left({\tilde{g}}_{i},{\tilde{q}}^{*}\right)\}\cdot \pi_{T}\left(T_{q}\right)\cdot Q_{T}\left(T_{q}^{\prime }|T_{q}\right)}. \end{array}$$

Note that, for simplicity, we use the same prior *π*_*T*_ and proposal $Q_{T}$ for each *T*_*i*_, *i* = 1, …, *C* and *T*_*q*_, but this is not required. Lastly, the algorithm updates $\sigma_{1}^{2}$ by drawing directly from an inverse-gamma distribution with shape parameter $\frac{1}{2}C\cdot N+a$ and scale parameter $\frac{1}{2}\sum_{i=1}^{C}\text{SSE}\left({\tilde{g}}_{i},{\tilde{q}}^{*}\right)+b$.

## Simulation study

**Fig 2:**
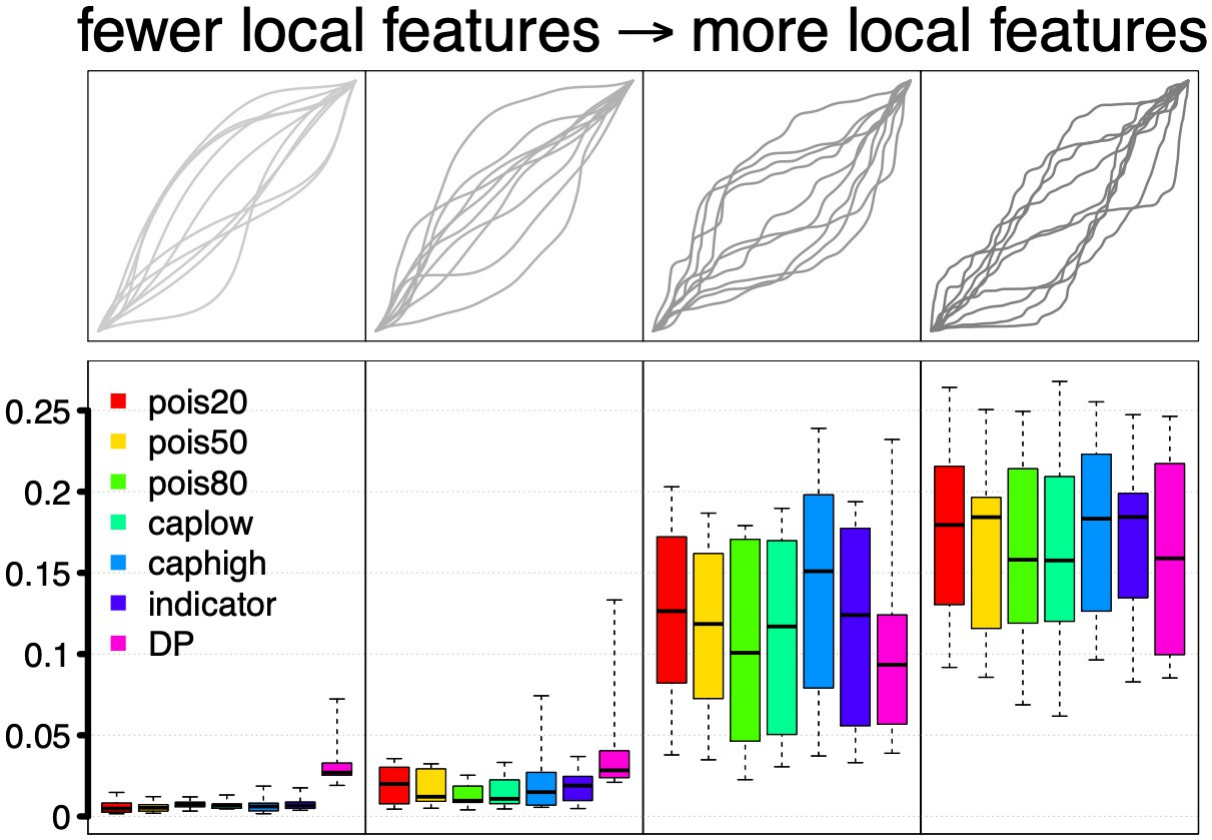
Pairwise simulation results. Top: Five sets of ten true warping functions with different amount of local features. Bottom: FR distance-based performance of different models for estimating the true warping functions in each set; smaller distance indicates better performance.

We first assess the performance of the proposed pairwise Bayesian registration model via a simulation study. We simulate two observed functions, *f*_1_ and *f*_2_, both of which are warped versions of a template function, *f*(*t*) = sin(4*πt*^2^) [2]. The two observed functions are constructed such that *f*_1_ = *f*_2_ ∘ *γ*_*true*_ where the true warping *γ*_*true*_ is randomly generated. We then apply our model to register *f*_2_ to *f*_1_ to obtain *γ*_*est*_, an estimate of the true warping, and compare *γ*_*est*_ to *γ*_*true*_. We use five sets of true warping functions, which are shown in Fig 2. Each set contains ten warpings and has varying degrees of local features: set (2) shown in the second panel in the top row contains warping functions that are very smooth with no “small wiggles,” whereas set (5) (fifth panel in the top row) contains warping functions that are “very wiggly.” Set (1) contains piecewise linear warping functions. We compare the proposed method to the model in [11], with the recommended setting of using 20 basis functions (we refer to this model as *M20*).

### Prior Specification

We now discuss different choices for the prior distributions for the pairwise registration Model (1). We use an inverse-gamma distribution with *a* = 0.1 and *b* = 0.1 for $\sigma_{1}^{2}$. For the Gaussian process prior of the warping parameter *g*, we must specify the covariance operator $C_{g}$ by its eigenpairs ${\left\{(b_{i},\lambda_{i}^{2})\right\}}_{i\geq 1}$. We use the Fourier basis functions $b_{i}\left(t\right)\in {\left\{\sqrt{2}\;\text{sin}\left(2i\pi t\right),\;\sqrt{2}\;\text{cos}\left(2i\pi t\right)\right\}}_{i\geq 1}$ and set $\lambda_{i}^{2}=4^{2}\cdot i^{-1.2}$. The constant 1.2 in this expression controls the decay rate of the Fourier series. If it is desirable to put more prior weight on warping functions with many local features, one can choose a smaller constant so that the higher frequency Fourier basis elements are weighted more (this constant should be greater than 1 to satisfy $\sum \lambda_{i}^{2}<\infty$). For the random truncation *T*, we use a few different priors based on which truncation mechanism is used, as described below.

**Truncation mechanism (1)**: There are multiple possible prior choices for the number of basis functions. First, we consider three Poisson priors, truncated to the domain [1, *M*_*max*_ = 200], with means equal to 20 (*pois20*), 50 (*pois50*) and 80 (*pois80*). These priors reflect beliefs about the smoothness of the warping functions. For instance, if the prior belief is that the warping function is relatively smooth and the phase variation should only relate to the general shape of the observed functions, a prior with a smaller mean should be chosen. Second, we consider two discrete uniform prior distributions on [30, *M*_*max*_ = 200] (*caplow*) and [1, 50] (*caphigh*), respectively. These two priors enforce a maximum (minimum) level of smoothness of the warping functions via the restriction that *M* ≥ 30 (*M* ≤ 50), but are non-informative in the sense that any *M* between 30 and *M*_*max*_ = 200 (1 and 50) is equally likely.

**Truncation mechanism (2)**: We choose a uniform prior distribution on the sequence of on-off switches for the basis functions (*indicator*). This prior is non-informative on the shape of the warping functions since any of the basis elements in the sequence are equally likely to be switched on or off.

The flexibility of prior choices for the random truncation is an important benefit of the proposed model, in comparison to existing models with fixed truncation. In practice, if one wishes to register the general shapes of the functions without altering small, local features, a prior that puts more weight on smoother functions (i.e., truncation mechanism (1) with Poisson with a small mean or a uniform with a small upper bound) should be chosen. If one has no strong prior opinion and wants the posterior to be mostly informed by the data, a less informative prior (i.e., truncation mechanism (2) with uniform for the on-off indicators) should be chosen. At the same time, the model is robust to the prior choices of the decay rate of the Fourier basis and the model variance $\sigma_{1}^{2}$ (see the S1 File for a sensitivity analysis).

### Implementation details

At the implementation stage, both the observed functions and the parameters are stored on a grid of size 200, which is the same as *M*_*max*_, the number of stored basis functions. We first perform pairwise registration for each set of warping functions using the deterministic Dynamic Programming (DP) algorithm, which is implemented in the R package *fdasrvf* [27]. We use the DP estimate to initialize the MCMC sampling algorithm for the proposed Bayesian model. We note that, while DP offers a good starting point and allows the chain to mix faster, the performance of the MCMC algorithm is independent of the starting point in the long run. Examples with different starting points are included in the S1 File. For the warping parameter *g*, we use a 3-mixture *pCN* proposal with jump sizes *β*_*z*_ = (0.5, 0.05, 0.0001) and corresponding proposal probabilities *p*_*z*_ = (0.3, 0.3, 0.4). For truncation mechanism (1), the proposal for *M* is a 10-step random walk where *M*′ stays at the current value or moves forward or backward by up to 10 steps (with the probability for each move (*p*_0_, *p*_1_, …, *p*_10_) ∝ (1, 0.5, 0.445, …, 0.001), where 0.5, 0.445, …, 0.001 is an equally spaced decreasing sequence of length 10). For truncation mechanism (2), the proposed state *χ*′ is generated by switching up to five randomly selected indicators. We use the *choose-k* proposal because we notice that it tends to have faster convergence than the *on-or-off* alternative.

### Results

To evaluate performance, we calculate the Fisher-Rao distance between *γ*_*true*_ and *γ*_*est*_ [24]:

$$\begin{array}{c} d_{FR}\left(\gamma_{true},\gamma_{est}\right)=\;{\text{cos}}^{-1}\left(\left\langle \psi_{true},\psi_{est}\right\rangle \right)=\;{\text{cos}}^{-1}(\int_{0}^{1}\psi_{true}\left(t\right)\psi_{est}\left(t\right)dt), \end{array}$$ (13)

where the posterior mean is used to construct *γ*_*est*_ for the Bayesian models. The results are shown in Fig 2. Importantly, we see that the *M20* model proposed in [11] does not perform well compared to the proposed random truncation or random indicator models when the true warping functions have many local features. While *M* can be fixed to a larger value, our model has the advantage that the value of *M* does not need to be decided a priori and, instead, is informed by the data. Comparing to DP, we notice that when *γ*_*true*_ has fewer local features (sets (2) and (3)), the proposed Bayesian models perform better irrespective of the prior distribution for the random truncation *T*. When *γ*_*true*_ has more local features (sets (4) and (5)), models *pois20* and *caphigh* perform worse, as expected, since they impose strong prior constraints on the maximum smoothness of the warping function. For the set of linear functions, DP performs better than the Bayesian models. This is not surprising since DP performs registration by solving a minimization problem in a piecewise linear fashion. On the other hand, the Fourier basis used for the Bayesian models are not piecewise linear. We also note that, while we compare DP with the Bayesian methods numerically using the FR distance, they are fundamentally different approaches. DP is optimization-based and the algorithm is very fast, but it has some known limitations: (1) it is not easy to enforce restrictions on the parameter space, (2) while regularization can be imposed by adding a term to the cost function, the choice of the regularization parameter is difficult in practice, (3) performance of the algorithm heavily depends on the discretization and neighborhood size settings, and (4) there is no prescribed approach for uncertainty quantification. In comparison, model-based approaches are more flexible and provide a more principled exploration of the parameter space.

**Fig 3:**
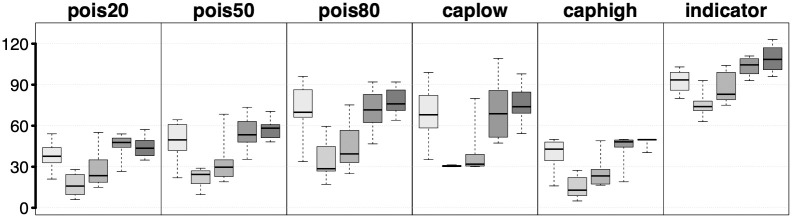
Posterior mean of the number of (active) basis functions for different registration models in the pairwise simulation study. The five boxplots in each panel correspond to the five sets of true warping functions (top row of Fig 2).

**Fig 4:**
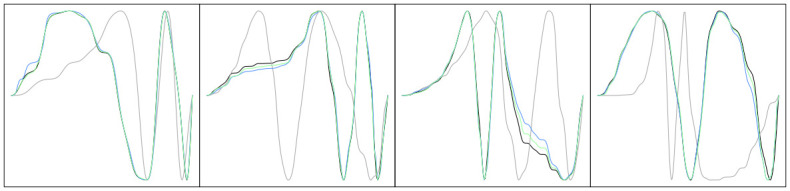
Four examples of pairwise Bayesian registration with priors *caplow* and *caphigh*. In each panel, we show *f*_1_ (black), *f*_2_ (grey), *f*_2_ ∘ *γ*_*est*,*caplow*_ (green) and *f*_2_ ∘ *γ*_*est*,*caplow*_ (blue). The estimated warping functions correspond to posterior means.

We also examine the posterior means of the number of active basis functions in Fig 3. Based on this result, we highlight two additional advantages of the proposed method compared to the model in [11]. First, inference on the level of smoothness of the warping function is clearly informed by the underlying data. We see that when the true warping functions, and consequently the observed functions, have more local features, the (posterior mean) number of active basis functions is larger regardless of the prior chosen for the random truncation *T*. Note that the set of linear functions requires more basis functions due to the sharp turns at the break points. Second, one can flexibly incorporate constraints on the level of smoothness via this prior. For example, the model *pois20* generates fewer active basis functions than the model *pois80*, regardless of the level of smoothness in the observed functions. The model *caplow* results in estimated warping functions based on at least 30 basis elements even when the true warping function is very smooth. On the other hand, the model *caphigh* results in estimated warping functions based on at most 50 basis elements even when the true warping function has many local features. Fig 4 shows four examples that compare registration performance between the Bayesian models *caplow* and *caphigh*. In each example, we display *f*_2_ (grey), *f*_1_ = *f*_2_ ∘ *γ*_*true*_ (black), *f*_2_ ∘ *γ*_*est*,*caplow*_ (green) and *f*_2_ ∘ *γ*_*est*,*caphigh*_ (blue). The estimated warping functions are obtained using the posterior means in each case. It is clear that the model *caplow* allows for registration of more local features than the model *caphigh*.

## Applications

In this section, we apply the proposed multiple function Bayesian registration model to the six datasets displayed in Fig 1. They arise in five different application domains:

1. **Right knee flexion and pelvis right roll**. This data is comprised of two gait variables (measurements taken by markers as participants walk) for *C* = 12 participants. We obtained the data from the online supplementary material of [28]. Each gait cycle is linearly scaled such that they are observed on the same time interval, i.e., the *x*-axis can be interpreted as percentage of one gait cycle. For more information on the role of registration in gait cycle analysis see, e.g., [10, 11, 29]
2. **Growth rates**. We use the growth rate functions of *C* = 54 girls from the Berkeley growth study [1], available in the R package *fda* [30]. This dataset is widely used to assess registration performance.
3. **Pinch force**. Each function records the pinch force exerted by the thumb and forefingers during a brief squeeze. These measurements were collected for *C* = 20 test subjects [2, 12, 31] and the full dataset is available in the R package *fda*. The starting time of the pinch as well as the time spent to reach the maximum force are different across test subjects, necessitating a registration step to account for this temporal variation prior to further analysis.
4. **Neural spike trains (sequence of electrical pulses sent by the neurons to the brain)**. The dataset is comprised of *C* = 10 smoothed neural spike train functions; see [9] for a detailed description. This dataset is analyzed in multiple papers, including [24, 32–36], with a particular interest in function registration.
5. **Handwriting samples**. We use the *x*-coordinates of *C* = 50 replicates (generated by a single person) of handwritten Chinese characters for ‘statistical science’ [6]), available in the R package *fda*. This dataset is used in [5] as an application of function registration.

### Prior specification

The covariance operator in the Gaussian process prior of the warping functions, $C_{g}$, and the inverse-gamma prior for the model variance, $\sigma_{1}^{2}$, are identical to the priors specified in the pairwise registration simulation study. The covariance operator in the Gaussian process prior for the template function, $C_{q}$, is specified by the Fourier basis with corresponding eigenvalues $\lambda_{i}^{2}=\sigma_{q}^{2}\cdot i^{-1.2}$, where $\sigma_{q}^{2}$ is fixed based on the scale of the observed functions. Using the Fourier basis to represent the template function is especially suitable when the observed functions are periodic on [0, 1], i.e., the gait cycle variables. For other datasets, we set the Fourier period to 2. As shown in the simulation study, these prior choices yield good registration results across different shapes of observed functions; on the other hand, the model is robust to alternative prior choices for these model parameters.

The random truncation is specified by truncation mechanism (1). For both the warping functions and the template, we use a discrete uniform prior on [5, *M*_*max*_ = 200] for the number of basis functions *M*. This prior is non-informative and allows us to evaluate registration performance that is primarily driven by the data. Intuitively, the chosen range, [5, *M*_*max*_ = 200], ensures that the warping functions have a minimum level of complexity (as captured by the first five Fourier basis elements), but are allowed to have as many local features as possible (since the observed functions are evaluated on a discretized grid of size 200). In practice, this is a suitable approach when one does not have strong prior information for the warping parameter and does not wish to restrict the level of shape-alteration during the registration process. For the proposals, we want to have a variety of small and intermediate jump sizes to explore the parameter space thoroughly. To that end, we use a *pCN* proposal with *β*_*z*_ values equally spaced between 0.001 and 0.0001 for the functional parameters and use a 1-step random walk proposal for the number of basis functions. Convergence is visually monitored by checking the trace plots of the log-likelihood. Trace plots for two of the datasets are provided in the S1 File.

### Results

**Fig 5:**
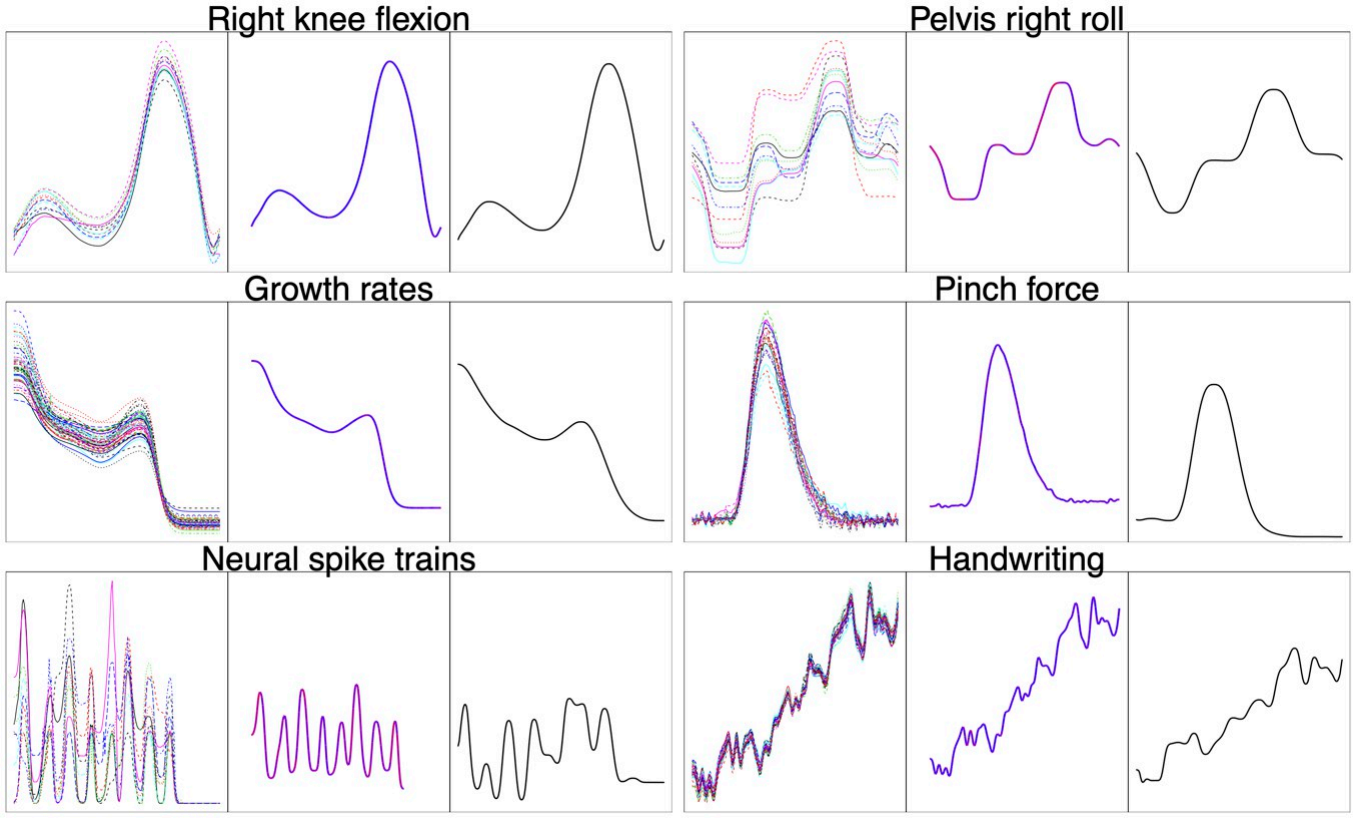
Registration results for six real datasets. The original observed functions for each dataset can be found in Fig 1. Here, we display the registered functions (left panel), the estimated template function using the proposed random truncation model (middle panel), with the color corresponding to the pointwise standard deviation (red—larger standard deviation and more uncertainty; blue—smaller standard deviation and less uncertainty), and the estimated template function using the M20 model (right panel). The warping and template functions used to perform registration are estimated using the posterior means.

For comparison, we also perform registration with the *M20* model [11]. Registration results for the six different datasets under consideration are shown in Fig 5. In each panel, we show the registered data (left) with respect to the estimated template (middle). For the estimated template, we also visualize the level of uncertainty (as measured by the posterior pointwise standard deviations, standardized by the scale of the original data; blue = small standard deviation, red = large standard deviation). We see that, when the observed functions have very different shapes (e.g., neural spike trains), the estimated template tends to have more uncertainty. In the right panel, we plot the template function estimated using the *M20* model. We see that the proposed method is better at producing a template function that resembles the shape of the original functions. For instance, the template estimated by the *M20* model does not have the small wiggles at both ends of the pinch force curves, and the *M20* model cannot recover a good template for the handwriting curves. This, again, shows the limitation of the model proposed in [11], where the number of basis functions can be mis-specified. In comparison, the proposed model uses 178 basis functions (posterior average) to estimate the template function of the pinch force dataset and 196 basis functions for the handwriting dataset. When the observed functions are relatively smooth, the posterior of the template function reflects that by using fewer basis functions (e.g., 55 and 33 for the two gait datasets). This shows the data-informative feature of the proposed method.

**Fig 6:**
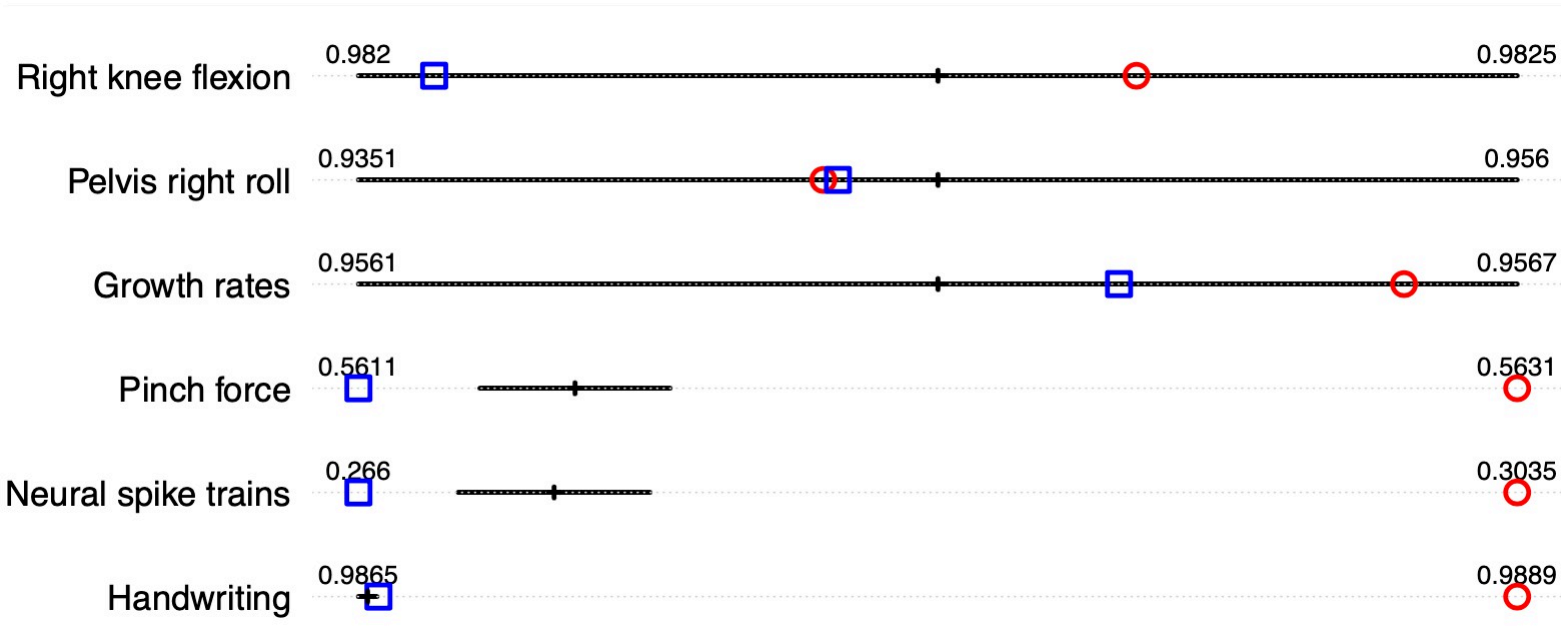
IPC values for six real datasets after registration. Smaller value means better alignment. The black line corresponds to the proposed approach (specifically, this is a 95% credible interval of the IPC values constructed using 200 randomly sampled posterior draws); the red round dot corresponds to the M20 model; the blue square corresponds to DP. Since the IPC value for each dataset is plotted on a different scale (and the values are not directly comparable across different datasets), we display the numeric values of the end points as a reference.

For a quantitative assessment of registration results, we report the inverse of pairwise correlation (IPC) [11, 15], calculated using $\text{IPC}=\frac{\sum_{i\neq j}r\left(f_{i},f_{j}\right)}{\sum_{i\neq j}r\left({\tilde{f}}_{i},{\tilde{f}}_{j}\right)}$, where *r*(⋅, ⋅) is the pairwise Pearson’s correlation, *f* are the observed functions, and $\tilde{f}$ are the registered functions. The IPC values corresponding to registration performance of different models are shown in Fig 6. Overall results are comparable across different models when the observed functions are relatively smooth. The *M20* model notably does not perform as well when the observed functions have many local features (e.g. pinch force, spike trains, and handwriting data) due to an under-specification of the number of basis functions. Compared to DP, the proposed Bayesian model achieves similar performance for all datasets (in the case of multiple function registration, DP aligns each function to an estimated template function in a pairwise manner; see [24] for details). Another numerical criterion (Sync) based on the $L^{2}$ distance between the registered functions shows similar results and is discussed in the S1 File.

**Fig 7:**
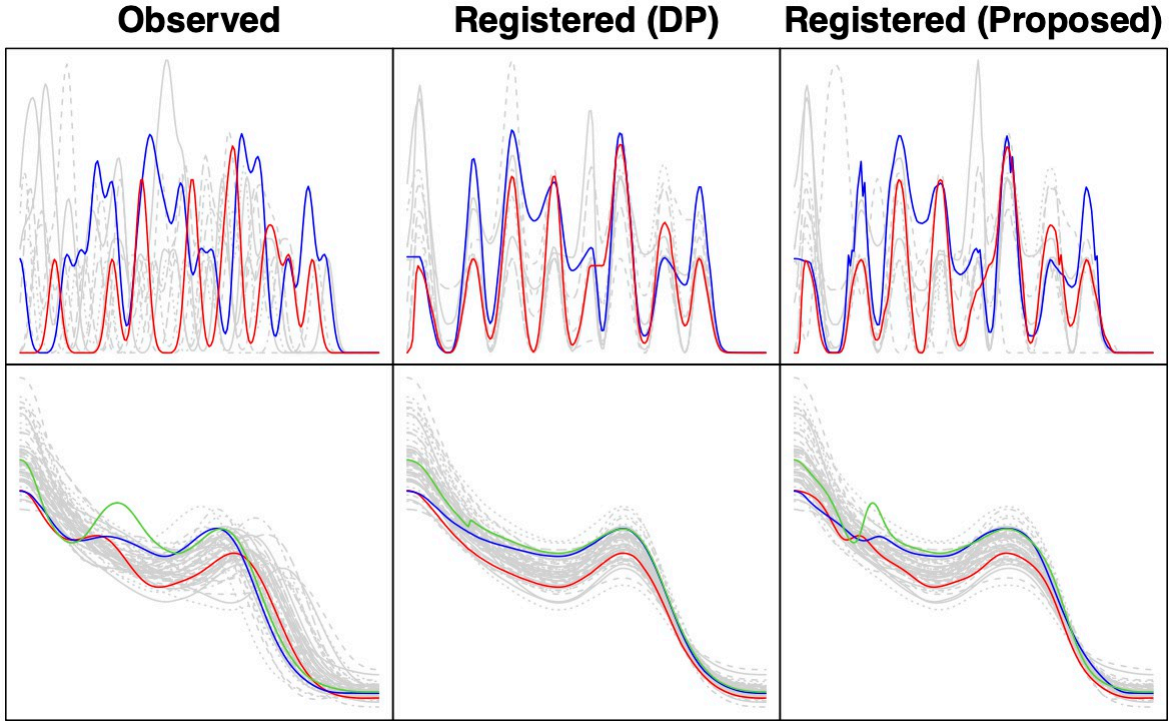
Registration results highlighting two neural spike trains (top) and three growth rate functions (bottom).

We note that the IPC (and Sync) values are based only on the correlation or the distance between the registered functions and do not take into account how well the shapes of the functions are preserved. It also only accounts for the quality of the template indirectly via the registered functions. As we have shown in Fig 5, the *M20* model does not recover the shape of the template function as well as the proposed method. On the other hand, DP sometimes does not preserve the original shapes of the observed functions after registration. For instance, we highlight two neural spike trains in the top row of Fig 7. The original observed curves are shown in the left panel; we see that one of the curves (blue) exhibits more local features than the other (red). Despite a good registration result (middle panel), DP has smoothed out the unmatched local features in the blue curve. In contrast (right panel), the proposed Bayesian model preserves the original shapes of the two curves better. In fact, when the observed functions have different numbers of features, e.g., local extrema, the random truncation component of the Bayesian model enables one to detect this pattern. The two highlighted neural spike trains were identified, because their corresponding warping parameters have the largest posterior means for the number of basis functions. An estimated warping function represented by a large number of basis elements signals that the observed function has been altered a lot in its local features after registration, likely due to some unmatched features being “squeezed,” as evident in this example.

In some applications, it is not desirable to alter the shapes of the observed functions too much. The proposed Bayesian model offers a flexible way to control how much shape alteration is allowed via the prior distribution of the random truncation parameter. Specifically, shape-alteration during the registration process will be limited if the prior puts very small or zero probability on large values of the number of basis functions. We show an example of this for the growth rate functions in the bottom row of Fig 7. We again highlight three observed functions in the left panel. They all exhibit two modes while most of the other functions in the data have only one mode. After registration using DP (middle panel), the bimodal pattern in the highlighted functions is no longer obvious. As a result, one might overlook the fact that these individuals have two growth spurts rather than the more common pattern of one pubertal growth spurt. To limit the level of shape alteration, we apply the proposed Bayesian registration model with a restrictive discrete uniform prior on [1, 10] for random truncation for the warping parameters. In this case, the prior limits the number of basis functions to be at most 10. The corresponding results (right panel) show that the bimodal feature of the highlighted growth rate functions is much better preserved after registration.

## Summary

We develop Bayesian models for pairwise and multiple function registration. These models build on existing Bayesian registration techniques which assign Gaussian process priors to the warping function, after a sequence of function space transformations. When building the registration models, the functional parameter is represented via an infinite sequence of basis functions, but at the implementation stage, it is necessary to truncate this sequence. Our main contribution lies in the randomization of this truncation process. This is done by introducing a new random truncation parameter that controls how many or which basis functions are used to represent the functional parameters. The resulting Bayesian models can then explore the full parameter space instead of a small subset of a truncated parameter space.

In practice, there are three main benefits of the proposed method compared to models where the truncation mechanism is fixed. First, the posterior distribution on the truncation parameter is informed by both the data and the prior, and one does not have to choose the truncation a priori, thus avoiding possible mis-specifications of the truncation parameter. Second, one can put restrictions on the registration process by using a restrictive prior for the truncation parameter, which controls how much shape alteration is allowed. For instance, as we have shown in the growth rate example, by limiting the number of basis functions to be at most 10 for the warping function parameter, we retain important features (one or two growth spurts) in the registered growth rate functions. Third, the new models also allow us to make inference on how much truncation has occurred. This can help detect when an observed function has undergone a significant shape change during registration, especially in its local features. We demonstrate the aforementioned advantages of the proposed approach through a simulation study and multiple real datasets.

In addition, the proposed modeling framework is very flexible. In the case of multiple function registration, one can use a different prior for each of the warping functions, corresponding to each of the observed functions, and for the template function, e.g., one can use non-informative priors for the warping functions and a restrictive prior for the template function that would constrain the template to have a smooth shape with limited local features. This enables fine-tuning of the models based on the application of interest.

The Metropolis-within-Gibbs algorithm we use to sample from the posterior distribution is efficient in the sense that the computational cost is largely unaffected by the size of the grid on which the functions are observed. The proposals for the functional and random truncation parameters further allow much flexibility in the jump sizes for exploring the parameter space. On the other hand, a drawback of the algorithm is that it is not informed by the likelihood. As a result, in some applications, convergence can be slow; for example, based on trace plots, the real datasets considered in the Applications section require at least 2 × 10^5^ iterations and can take more than 10^6^ iterations (trace plots for two datasets are given as examples in the S1 File; computation time for registering 10 functions is about 100 minutes per 10^5^ updates). While this is not surprising, because the algorithm is exploring a very large parameter space, a possible future research direction is to design algorithms with faster convergence rates, by using likelihood-informed proposals or adaptive proposals with jump sizes automatically tuned by acceptance rates.

## Supporting information

S1 File Supplementary material. This pdf file serves as an appendix to the main manuscript and includes: 1) additional derivations; 2) trace plots for two real data examples; 3) Sync values for assessing registration performance for the six real datasets in Section; and 4) a discussion of model sensitivity.
(PDF)

S2 File Code and data. The code folder includes R code to perform pairwise and multiple function registration. Datasets used in the manuscript are also included as .RData files. A readme file is included to provide instructions.
(ZIP)
