# Supplementary material for: Bayesian function registration with random truncation
Source: PLoS One. 2023 Jul 7;18(7):e0287734. doi: 10.1371/journal.pone.0287734 (PMC10328359; doi:10.1371/journal.pone.0287734)
Supplement: S1 File — This pdf file serves as an appendix to the main manuscript and includes: 1) additional derivations; 2) trace plots for two real data examples; 3) Sync values for assessing registration performance for the six real datasets in Section; and 4) a discussion of model sensitivity. (PDF) [file pone.0287734.s001.pdf]

Supplementary Material for “Bayesian Function Registration with Random Truncation”

Yi Lu[1¤a], Radu Herbei[2¤b], Sebastian Kurtek[2*¤b],

1 Mathematics and Computer Science Department, Drew University, Madison, New Jersey, the United States of America
2 Department of Statistics, The Ohio State University, Columbus, Ohio, the United States of America

¤a Current Address: Mathematics and Computer Science Department, Drew University, Madison, New Jersey, the United States of America
¤b Current Address: Department of Statistics, The Ohio State University, Columbus, Ohio, the United States of America
*kurtek.1@stat.osu.edu

More on Function Space Transformations

Motivation for Transforming the Observation Space

Suppose we observe two functions f_1 and f_2 and aim to perform pairwise registration. As described in the main article, we transform both f_1 and f_2 to their square root velocity functions (SRVFs) q_1 and q_2. There are two main reasons for this transformation. First, we wish to avoid registration asymmetry. It can be easily verified that

$$\inf_{\gamma_1,\gamma_2\in\Gamma} \|f_1 \circ \gamma_1 - f_2 \circ \gamma_2\|_2 \neq \inf_{\gamma_1\in\Gamma} \|f_1 \circ \gamma_1 - f_2\|_2 \neq \inf_{\gamma_2\in\Gamma} \|f_1 - f_2 \circ \gamma_2\|_2, \tag{1}$$

where $\|\cdot\|_2$ is the $\mathbb{L}^2$ norm. In other words, registering f_2 to f_1 can generate a very different result from registering f_1 to f_2. On the other hand, under the SRVF representation, we have the desired symmetry, i.e.,

$$\inf_{\gamma_1,\gamma_2\in\Gamma} \|(q_1, \gamma_1) - (q_2, \gamma_2)\|_2 = \inf_{\gamma_1\in\Gamma} \|(q_1, \gamma_1) - q_2\|_2 = \inf_{\gamma_2\in\Gamma} \|q_1 - (q_2, \gamma_2)\|_2 \,. \tag{2}$$

Second, the registration problem in Equation (1) suffers from the so-called pinching effect where the distance between any two functions after warping can become arbitrarily small [1]. This issue does not arise in the registration problem in Equation (2).

Motivation for Transforming the Parameter Space

The parameter space $\Gamma = \{\gamma : [0, 1] \mapsto [0, 1] \mid \gamma(0) = 0, \gamma(1) = 1,\ 0 < \gamma' < \infty\}$ is nonlinear, and we aim to apply transformations that can simplify the complicated geometry of this space. For each $\gamma \in \Gamma$, we first apply the SRVF transformation $Q(\gamma)(t) = \sqrt{\gamma'(t)}$ and denote the resulting function by $\psi(t)$. The space of ψ is $\Psi \equiv \{\psi : [0, 1] \mapsto \mathbb{R}^+ \mid \|\psi\|_2 = 1\}$, the positive orthant of the unit sphere in the Hilbert space $\mathbb{L}^2([0, 1])$. Borrowing standard tools from differential geometry, we can map (unwrap) the unit sphere onto the tangent space at $1 \in \Psi$, defined as $T_1(\Psi) = \left\{g : [0, 1] \mapsto \mathbb{R} \mid \langle g, 1\rangle = \int_0^1 g(t)dt = 0\right\}$, using the inverse exponential map, defined in the main article.

Working in the transformed space $T_1(\Psi)$ rather than Γ has two important advantages. First, the Fisher-Rao metric on Γ can be easily calculated on Ψ as $d_{FR}(\gamma_1, \gamma_2) = \cos^{-1}(\langle\psi_1, \psi_2\rangle) = \cos^{-1}\left(\int_0^1 \psi_1(t)\psi_2(t)dt\right)$. This enables efficient summarization of the posterior (e.g., computation of the mean) and quantification of the difference between an estimated γ and a true γ in simulation studies. Second, the tangent space $T_1(\Psi)$ is a linear space and its elements can be represented as linear combinations of standard basis functions. Third, this transformation allows us to define a standard Gaussian process probability model on this space.

Derivation of ρ in Equation 8 of the Main Article

Suppose $g \in \mathcal{H}$ and $T \in \mathcal{T}$. Consider the measurable space $(\mathcal{H} \times \mathcal{T}, \mathcal{B}_\mathcal{H} \otimes \mathcal{B}_\mathcal{T})$. The prior distribution for (g, T) is a measure on this space defined as $\mathcal{P}_0(A) = \mathcal{P}^*(A \cap B)/\mathcal{P}^*(B)$, where $A \subset \mathcal{H} \times \mathcal{T}$ is a measurable set of (g, T), B is the domain of (g, T), $\mathcal{P}^*(B)$ is a normalizing constant, and the unrestricted measure $\mathcal{P}^*$ is defined via measurable rectangles, as $\mathcal{P}^*(A_1 \times A_2) = \mu_0(A_1) \cdot \tau_T(A_2)$, $A_1 \in \mathcal{B}_\mathcal{H}$, $A_2 \in \mathcal{B}_\mathcal{T}$, where μ_0 is the Gaussian

prior measure for g and τ_T is the prior measure for T. Now, the target measure $\mathcal{P}$ is the full conditional distribution of (g, T), which is dominated by $\mathcal{P}_0$, with the Radon-Nikodym derivative given by the likelihood, i.e.,

$$\frac{d\mathcal{P}}{d\mathcal{P}_0}(g, T) \propto L(g, T, \sigma_1^2).$$

Let (g', T') be the proposed value and (g, T) be the current value. The proposal kernel is given by $q_{(g',T')}(A_1 \times A_2) = q_{g'}(A_1) \times q_{T'}(A_2), A_1 \in \mathcal{B}_{\mathcal{H}}, A_2 \in \mathcal{B}_{\mathcal{T}}$, where $q_{g'}(\cdot)$ is induced by the pCN proposal and $q_{T'}(\cdot)$ is induced by $\mathbb{Q}_T(\cdot|T')$. Under this set up, the Metropolis-Hastings acceptance ratio is given by

$$\begin{aligned}
\rho &= \left\{\frac{d\mathcal{P}}{d\mathcal{P}_0}(g', T') \cdot \frac{dq_{(g',T')}}{d\mathcal{P}_0}(g, T)\right\} \left\{\frac{d\mathcal{P}}{d\mathcal{P}_0}(g, T) \cdot \frac{dq_{(g,T)}}{d\mathcal{P}_0}(g', T')\right\}^{-1} \\
&= \left\{L(g', T', \sigma_1^2) \cdot \frac{dq_{(g',T')}}{d\mathcal{P}_0}(g, T)\right\} \left\{L(g, T, \sigma_1^2) \cdot \frac{dq_{(g,T)}}{d\mathcal{P}_0}(g', T')\right\}^{-1}.
\end{aligned}$$

Next, we show that

$$\int_{A_1 \times A_2} \frac{dq_{(g',T')}}{d\mathcal{P}_0}(g, T) \cdot I\{(g, T) \in B\}\ d\mathcal{P}^* = \mathcal{P}^*(B) \cdot \int_{A_1 \times A_2} \frac{dq_{g'}}{d\mu_0}(g) \cdot \frac{dq_{T'}}{d\tau_T}(T)\ d\mathcal{P}^*.$$

$$\begin{aligned}
lhs &= \int_{(A_1 \times A_2) \cap B} \frac{dq_{(g',T')}}{d\mathcal{P}_0}(g, T)\ \mathcal{P}^*(dg \times dT) \\
&= \int_{A_1 \times A_2} \frac{dq_{(g',T')}}{d\mathcal{P}_0}(g, T) \cdot \mathcal{P}^*(B)\ \mathcal{P}_0(dg \times dT)\ (\textit{by the definition of } \mathcal{P}_0) \\
&= \mathcal{P}^*(B) \cdot q_{(g',T')}(A_1 \times A_2) \\
&= \mathcal{P}^*(B) \cdot q_{g'}(A_1) \times q_{T'}(A_2) \\
&= \mathcal{P}^*(B) \cdot \int_{A_1} \frac{dq_{g'}}{d\mu_0}(g)\ \mu_0(dg) \cdot \int_{A_2} \frac{dq_{T'}}{d\tau_T}(T)\ \tau_T(dT) \\
&= \mathcal{P}^*(B) \cdot \int_{A_1 \times A_2} \frac{dq_{g'}}{d\mu_0}(g) \cdot \frac{dq_{T'}}{d\tau_T}(T)\ d\mathcal{P}^*\ (\textit{by Fubini's theorem and the definition of } \mathcal{P}^*) \\
&= rhs.
\end{aligned}$$

In other words,

$$\frac{dq_{(g',T')}}{d\mathcal{P}_0}(g, T) \cdot I\{(g, T) \in B\} = \mathcal{P}^*(B) \cdot \frac{dq_{g'}}{d\mu_0}(g) \cdot \frac{dq_{T'}}{d\tau_T}(T)\ \ a.e.\ \mathcal{P}^*,$$

and it follows that

$$\begin{aligned}
\rho = &\left\{L(g', T', \sigma_1^2) \cdot \mathcal{P}^*(B) \cdot \frac{dq_{g'}}{d\mu_0}(g) \cdot \frac{dq_{T'}}{d\tau_T}(T) \cdot I\{(g', T') \in B\}\right\} \\
&\left\{L(g, T, \sigma_1^2) \cdot \mathcal{P}^*(B) \cdot \frac{dq_g}{d\mu_0}(g') \cdot \frac{dq_T}{d\tau_T}(T') \cdot I\{(g, T) \in B\}\right\}^{-1}.
\end{aligned}$$

Since the normalizing constant $\mathcal{P}^*(B)$ cancels out, the term $dq_{g'}/d\mu_0(g)$ is symmetric in g and g' for the pCN proposal ([2]), and $(g, T) \in B$ is always true for the current state, we have

$$\rho = \left\{L(g', T', \sigma_1^2) \cdot \frac{dq_{T'}}{d\tau_T}(T) \cdot I\{(g', T') \in B\}\right\} \left\{L(g, T, \sigma_1^2) \cdot \frac{dq_T}{d\tau_T}(T')\right\}^{-1}.$$

Furthermore, suppose densities with respect to the Lebesgue measure exist on $\mathcal{T}$, and denote by $\pi_T(\cdot)$ and $\mathbb{Q}_T(\cdot|T')$ the densities of τ_T and $q_{T'}$ respectively, we have

$$\rho = \frac{L(g', T', \sigma_1^2) \cdot \pi_T(T') \cdot \mathbb{Q}_T(T|T')}{L(g, T, \sigma_1^2) \cdot \pi_T(T) \cdot \mathbb{Q}_T(T'|T)} \cdot I\{(g', T') \in B\}. \ \square$$

Derivation of $a_{\chi,\chi'}$ in Equation 10 of the Main Article

$a_{\chi,\chi'} = \mathbb{Q}_\chi(\chi|\chi')/\mathbb{Q}_\chi(\chi'|\chi)$ where the proposal density has non-zero probabilities in the following cases:

$$\begin{cases} \mathbb{Q}_\chi(\chi' \ni M'_{on} = 2|\chi \ni M_{on} = 1) = 1/(M_{max} - 1) \\ \mathbb{Q}_\chi(\chi' \ni M'_{on} = M_{max} - 1|\chi \ni M_{on} = M_{max}) = 1/M_{max} \\ \mathbb{Q}_\chi(\chi' \ni M'_{on} = M_{on} - 1|\chi \ni M_{on} \neq 1, M_{on} \neq M_{max}) = 1/2 \cdot (1/M_{on}) \\ \mathbb{Q}_\chi(\chi' \ni M'_{on} = M_{on} + 1|\chi \ni M_{on} \neq 1, M_{on} \neq M_{max}) = 1/2 \cdot (1/(M_{max} - M_{on})). \end{cases}$$

Now, for $\chi \in \{\chi|M_{on} = 1\}$ and $\chi' \in \{\chi|M_{on} = 2\}$,

$$\frac{\mathbb{Q}_\chi(\chi|\chi')}{\mathbb{Q}_\chi(\chi'|\chi)} = \frac{1/2 \cdot 1/2}{1/(M_{max} - 1)} = (M_{max} - 1)/4.$$

For $\chi \in \{\chi|M_{on} = M_{max}\}$ and $\chi' \in \{\chi|M_{on} = M_{max} - 1\}$,

$$\frac{\mathbb{Q}_\chi(\chi|\chi')}{\mathbb{Q}_\chi(\chi'|\chi)} = \frac{1/2}{1/M_{max}} = M_{max}/2.$$

For $\chi \in \{\chi|M_{on} = k, k \neq 1, k \neq M_{max}\}$ and $\chi' \in \{\chi|M_{on} = k + 1\}$,

$$\frac{\mathbb{Q}_\chi(\chi|\chi')}{\mathbb{Q}_\chi(\chi'|\chi)} = \frac{1/2 \cdot 1/(k+1)}{1/2 \cdot 1/(M_{max} - k)} = (M_{max} - k)/(k + 1).$$

For $\chi \in \{\chi|M_{on} = k, k \neq 1, k \neq M_{max}\}$ and $\chi' \in \{\chi|M_{on} = k - 1\}$,

$$\frac{\mathbb{Q}_\chi(\chi|\chi')}{\mathbb{Q}_\chi(\chi'|\chi)} = \frac{1/2 \cdot 1/(M_{max} - k + 1)}{1/2 \cdot 1/k} = k/(M_{max} - k + 1). \ \square$$

Trace Plots

Trace plots of the log-likelihood for two real data examples in Section 6 of the main article are given in Fig 1.

Synchronization Coefficient Values

In the main article, we report the IPC (inverse of pairwise correlation) value to quantitatively assess registration performance for six real datasets (Applications section). Another metric, the synchronization coefficient (Sync) [3, 4], is calculated as

$$\text{Sync} = \frac{1}{C} \sum_{i=1}^{C} \frac{\|\tilde{f}_i - \frac{1}{(C-1)} \sum_{j \neq i} \tilde{f}_j\|^2}{\|f_i - \frac{1}{(C-1)} \sum_{j \neq i} f_j\|^2},$$

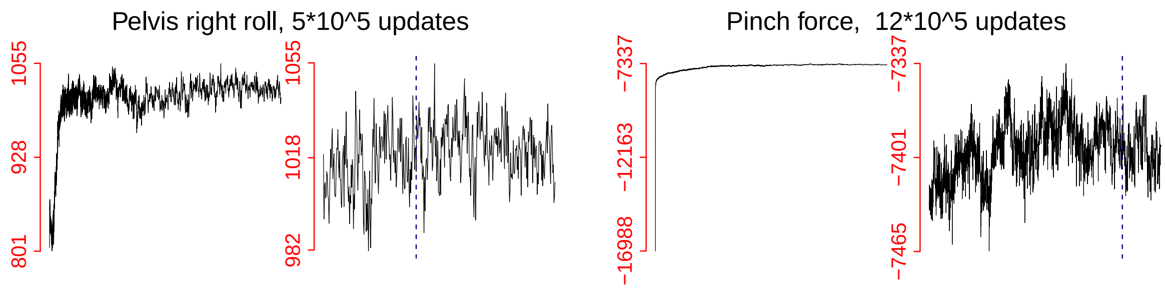

Figure 1: *Trace plots of the log-likelihood for two datasets. The panel on the left shows the entire chain and the panel on the right shows the second half of the chain. The dashed line indicates the end of the burn-in period.*

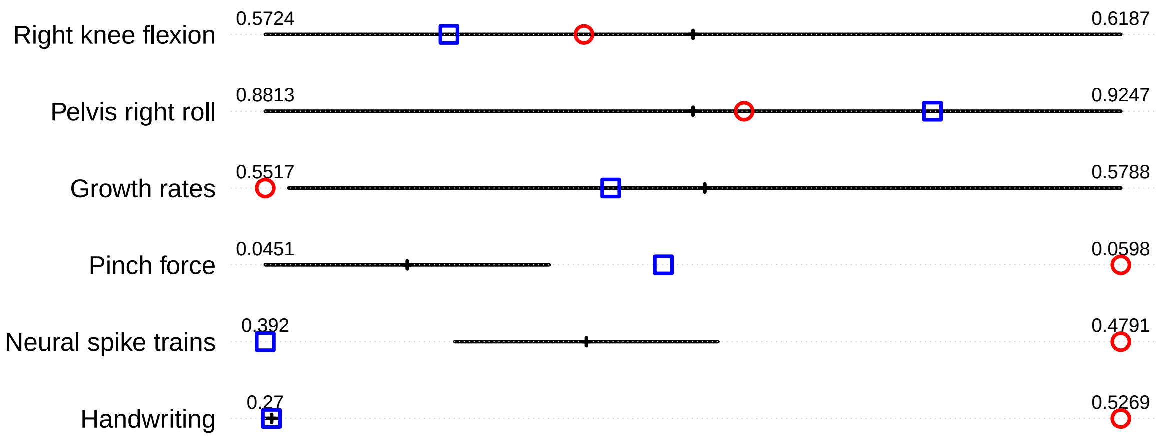

Figure 2: *Sync values for six real datasets after registration (smaller value means better alignment). The black line corresponds to the proposed approach (specifically, it is a* 95% *credible interval of the Sync values constructed using* 200 *randomly sampled posterior draws); the red round dot corresponds to the M20 model [4], while the blue square corresponds to dynamic programming. Since the Sync value for each dataset is plotted on a different scale (and the values are not directly comparable across different datasets), we display the numeric values of the end points as a reference.*

where C is the number of functions in the data, f are the observed functions, and $\tilde{f}$ are the registered functions. The Sync values after registering the six real datasets are shown in Fig 2. We note that the Sync criterion uses the $\mathbb{L}^2$ distance to measure alignment, and it suffers from the pinching effect (as discussed earlier in this Supplementary Material). In other words, it favors registration performance when some peaks have been "squeezed". In addition, the registered functions are invariant to a common warping (i.e., the template function is not uniquely identifiable) and the Sync criterion is highly sensitive to the shape of the recovered template (again, a shape with squeezed features will yield a lower Sync value). To avoid this issue, we center the registered functions such that the Karcher mean [5, 6] of their estimated warpings is the identity function. The reported Sync values (and IPC values in the main article) are calculated after this centering step.

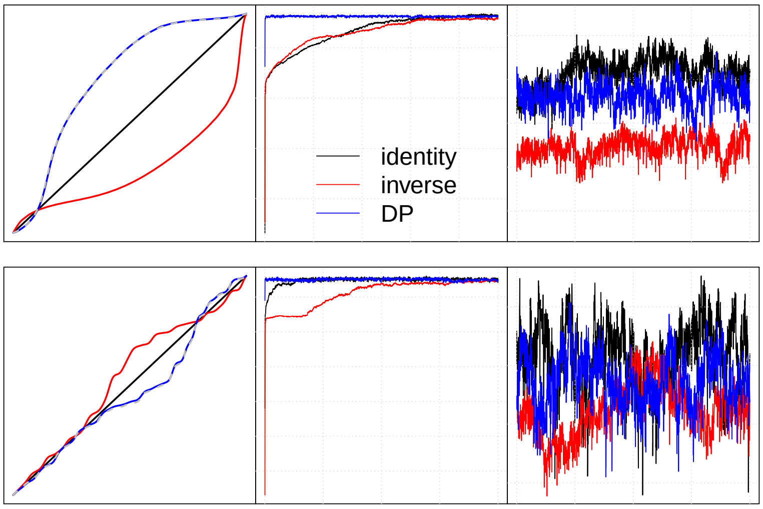

Figure 3: *Trace plots of the log-likelihood for two pairwise registration examples. The MCMC chain was initialized using different starting values: the identity warping function* $\gamma(t) = t$ *(identity); the inverse of the true warping function (inverse); and the DP estimated warping function (DP). These are shown in the left panels in black, red and blue along with the true warping function in grey (dashed). The middle panels show the log-likelihood of* 6×10^6 *updates (top) and* 12×10^6 *updates (bottom). The right panels show the log-likelihood of the last* 10^6 *updates.*

Alternative Implementations - Sensitivity Analysis

We change some of the implementation settings used in the main article to investigate model sensitivity. First, we look at convergence of the MCMC chain when the starting point is not informed by dynamic programming (DP). Specifically, we use two examples in the pairwise simulation study (one where the true warping function is relatively smooth and one where the true warping function has many local features). We perform registration with the model *pois50* (i.e., the prior for the number of basis functions is a Poisson distribution with mean 50). We initialize the MCMC chain with three different starting values: (1) the identity warping function $\gamma(t) = t$; (2) the inverse of the true warping function; and (3) the DP estimate (these are shown in the left panels of Fig 3). Trace plots of the log-likelihood are shown in the middle and right panels of Fig 3. We see that, while the DP estimate provides a good starting point, the performance of the MCMC algorithm, in the long run, does not depend on the starting point. In practice, all three starting points provide very good registration results after a long burn-in period (the estimated warping functions are visually identical). If using a "bad" starting point (e.g., one that is very far away from a posterior mode), we recommend choosing a proposal that has many varying jump sizes to ensure an efficient and thorough exploration of the large parameter space.

Second, we evaluate registration performance for the same two pairwise examples under different prior choices. The alternative settings and corresponding registration results are provided in Fig 4. We see that the model is robust to the choice of the decay rate of the Fourier basis. When the decay rate is larger (e.g., 3 instead of the default value of 1.2), the later Fourier basis functions have smaller coefficients in the expansion. In other words, the prior puts more probability on smoother functions, and, as a result,

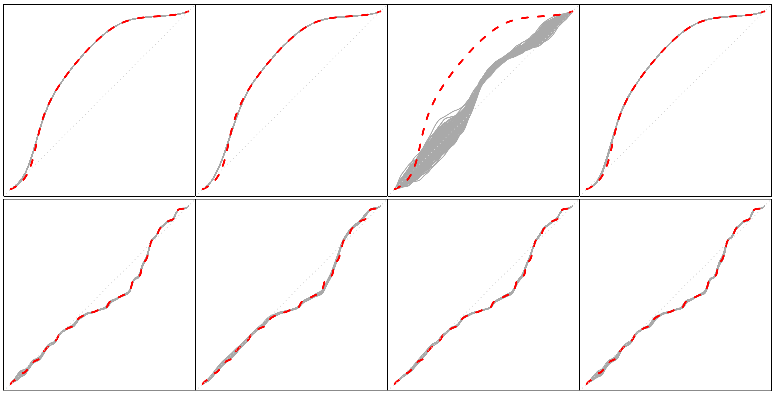

Figure 4: *Pairwise registration examples under different prior settings. In each panel, the red curve is the true warping function, and the grey curves are 200 random draws from the posterior. The two rows correspond to two different examples. For each example, we use four sets of prior settings: (left to right) 1. the decay rate of the Fourier basis for the prior of g is set to* 2 *(instead of* 1.2*); 2. the decay rate is set to* 3*; 3. the standard deviation in the prior of g is set to* 0.1 *(instead of 4); and 4. the hyperparameters for the inverse-gamma prior of the model variance are set to $a = 2$ and $b = 1$ (instead of 0.1 and 0.1).*

the estimated warping also appears smoother. The model is also robust to the choice of the hyperparameters of the inverse-gamma prior of the model variance σ_1^2. However, the model performs poorly when the standard deviation in the prior of g is too small. This is not surprising since, in this case, the prior for the warping function is very concentrated around the identity function and the posterior does not deviate too far from it.

References

[1] Marron JS, Ramsay JO, Sangalli LM, Srivastava A. Functional Data Analysis of Amplitude and Phase Variation. Statistical Science. 2015;30(4):468–484.

[2] Cotter SL, Roberts GO, Stuart AM, White D, et al. MCMC Methods for Functions: Modifying Old Algorithms to Make them Faster. Statistical Science. 2013;28(3):424–446.

[3] Cheng W, Dryden IL, Huang X. Bayesian Registration of Functions and Curves. Bayesian Anal. 2016;11(2):447–475.

[4] Lu Y, Herbei R, Kurtek S. Bayesian Registration of Functions with a Gaussian Process Prior. Journal of Computational and Graphical Statistics. 2017;26(4):894–904.

[5] Karcher H. Riemannian Center of Mass and Mollifier Smoothing. Communications on Pure and Applied Mathematics. 1977;30(5):509–541.

[6] Srivastava A, Wu W, Kurtek S, Klassen E, Marron JS. Registration of Functional Data Using Fisher-Rao Metric. arXiv preprint. 2011b;arXiv:1103.3817.
