# Supplementary material for: Bayesian function registration with random truncation
Source: PLoS One. 2023 Jul 7;18(7):e0287734. doi: 10.1371/journal.pone.0287734 (PMC10328359; doi:10.1371/journal.pone.0287734)
Supplement: S2 File — The code folder includes R code to perform pairwise and multiple function registration. Datasets used in the manuscript are also included as .RData files. A readme file is included to provide instructions. (ZIP) [file pone.0287734.s002.zip › code/readme.rtf]

R packages The first section of registration_functions.R will install the following R packages; it is recommended to install these packages first and comment out the “install.packages()” codes.caToolscodafdafdasrvfggplot2gridMASSMCMCpacknumDerivplotrixpurrrstatsPairwise registration1. open pw_registration.R2. make sure the working directory is set to the current folder 3. (optional) change the filename of saved results by specifying SAVE.NAME4. (optional) specify how long to run the chain by adjusting:	pw.sim.global.NCHAIN: length of the chain	pw.sim.global.burnin: length of the burn-in period	pw.sim.global.save.frequency: how much results should be saved (e.g., if pw.sim.global.save.frequency=2, then updates 1, 3, 5, etc. will be saved)5. (optional) specify prior and proposal distributions; the prior distribution used in this file corresponds to truncation mechanism (1) in the main manuscript (i.e., T=M=number of basis functions); the prior for M can either be poisson or uniform6. run all lines of codes; results will be saved to the folder “result”Multiple registration1. open the file mfr_registration.R2. make sure the working directory is set to the current folder  3. (optional) change the filename of saved results by specifying SAVE.NAME4. (optional) specify which dataset and which functions in the dataset to be registered by adjusting:	DATA: name of the dataset (choices: “gcflex”, “gcpel”, “growth”, “pinch”, “spike”, “writingx”, “writingy”; these datasets are formatted and stored in registration_data.RData; sources of each dataset are cited in the main manuscript.)	REGISTERINDEX: index of the functions within the dataset to be registered5. (optional) specify how long to run the chain by adjusting:	global.NCHAIN: length of the chain	global.burnin: length of the burn-in period	global.save.frequency: how much results should be saved6. run all lines of codes; results will be saved to the folder “result”
